# Measurement Error Affects Risk Estimates for Recruitment to the Hudson River Stock of Striped Bass

**DOI:** 10.1100/tsw.2002.865

**Published:** 2002-06-07

**Authors:** Dennis J. Dunning, Quentin E. Ross, Stephan B. Munch, Lev R. Ginzburg

**Affiliations:** New York Power Authority, 123 Main Street, White Plains, NY 10601, USA; Applied Biomathematics, 100 North Country Road, Setauket, NY 11733, USA

**Keywords:** measurement error, ecological risk assessment, recruitment, striped bass, Hudson River, adverse environmental impact, entrainment, Section 316(b), Clean Water Act, mitigation, sustainability

## Abstract

We examined the consequences of ignoring the distinction between measurement error and natural variability in an assessment of risk to the Hudson River stock of striped bass posed by entrainment at the Bowline Point, Indian Point, and Roseton power plants. Risk was defined as the probability that recruitment of age-1+ striped bass would decline by 80% or more, relative to the equilibrium value, at least once during the time periods examined (1, 5, 10, and 15 years). Measurement error, estimated using two abundance indices from independent beach seine surveys conducted on the Hudson River, accounted for 50% of the variability in one index and 56% of the variability in the other. If a measurement error of 50% was ignored and all of the variability in abundance was attributed to natural causes, the risk that recruitment of age-1+ striped bass would decline by 80% or more after 15 years was 0.308 at the current level of entrainment mortality (11%). However, the risk decreased almost tenfold (0.032) if a measurement error of 50% was considered. The change in risk attributable to decreasing the entrainment mortality rate from 11 to 0% was very small (0.009) and similar in magnitude to the change in risk associated with an action proposed in Amendment #5 to the Interstate Fishery Management Plan for Atlantic striped bass (0.006)— an increase in the instantaneous fishing mortality rate from 0.33 to 0.4. The proposed increase in fishing mortality was not considered an adverse environmental impact, which suggests that potentially costly efforts to reduce entrainment mortality on the Hudson River stock of striped bass are not warranted.

### INTRODUCTION

The U.S. Environmental Protection Agency (EPA) is required to issue regulations for implementing Section 316(b) of the Clean Water Act (CWA), 33 U.S.C. Section 1326(b). Section 316(b) provides that any standard established pursuant to Sections 301 or 306 of the CWA and applicable to a point source shall require that the location, design, construction, and capacity of the cooling-water intake structures reflect the best technology available (BTA) for minimizing adverse environmental impact (AEI). Early guidance provided by the EPA indicated that AEI occurs whenever there is entrainment or impingement of aquatic organisms resulting from the operation of a cooling-water intake structure[1]. However, this policy could require costly mitigation like a cooling tower that produces little benefit if an alternative definition of AEI is adopted, e.g., one based on populations. In such high-stakes cases, the degree of environmental protection and the associated cost should be reconciled with scientific data and methods[2].

Recently, the EPA began a process to update and formalize its early guidance for defining and assessing AEI. Factors that the EPA is considering include new approaches and tools developed since the early guidance was issued[3]. One of the tools being considered is ecological risk assessment. It is used to evaluate the likelihood that adverse ecological effects may occur or are occurring as a result of exposure to one or more stressors and includes an evaluation of uncertainty[4].

Two types of uncertainty affect risk assessments for populations[5]. One is intrinsic to the populations, reflecting natural variability in abundance. The other reflects variability in abundance due to sampling (i.e., measurement error). This distinction is not usually recognized in risk analyses of population extinction[6,7,8,9], but the distinction may be very important. Using very simplified and idealized numerical examples, Ferson and Ginzburg[5] demonstrated that failure to partition natural variability and measurement error could produce biased estimates of risk.

Large measurement errors, which are present in most fisheries data, result in substantial uncertainty in abundance estimates[10], often overwhelming effects of density dependence in stock-recruitment relationships[11]. To improve the credibility of scientific advice and to provide better information, measurement error must be considered explicitly[12]. Analysis of the effects of measurement error usually involves bootstrapping simulation studies because multiple, independent estimates of specific parameters, needed to estimate measurement error, are rarely available. For the Hudson River stock of striped bass, multiple, independent indices of abundance are available and measurement error can be estimated.

The effects of entraining fish, especially striped bass, at power plants operating on the Hudson River have been of considerable interest to regulators, electric utilities, and the public[13,14,15]. Currently, the New York State Department of Environmental Conservation (the Department) is reviewing applications to renew the State Pollutant Discharge Elimination System (SPDES) permits for power plants operating on the Hudson River at the Bowline Point, Indian Point, and Roseton sites. In the review process, the Department will consider the level of uncertainty that can be accommodated in making a decision on the SPDES permit renewals (i.e., what level of risk to the fishery resource is acceptable)[16]. The Department will also consider issuing consecutive SPDES permits covering a time horizon of up to 15 years as an alternative to issuing a single permit for a 5-year period[17].

The choice of time horizon can strongly affect both the outcome and reliability of risk assessments[18]. For shorter time horizons, the risks of alternative actions may not differ appreciably. In such cases, having estimates of measurement error would be less critical than for longer time horizons where measurement error may hide real differences in risk. Our objectives were to identify a measure of risk for the Hudson River stock of striped bass that could be used to evaluate the effects of entrainment mortality at the Bowline, Indian Point, and Roseton power plants, assess the effects of measurement error and time on the risk estimates, and compare the risks due to entrainment mortality with those due to increased fishing mortality recommended under Amendment #5 to the Interstate Fishery Management Plan for Atlantic striped bass.

### METHODS

We evaluated risk by examining changes in recruitment to the Hudson River stock of striped bass due to entrainment mortality. Indices of recruitment serve as input to the spawning stock model used by the Atlantic States Marine Fisheries Commission (ASMFC) to estimate future population levels and as an early warning signal to fishery managers[19]. The measure of recruitment accepted by the ASMFC is juvenile (age 0) abundance. However, we used abundance of age-1+ (i.e., yearling) striped bass in the Hudson River to represent recruitment because abundance estimates for juvenile striped bass in the Hudson River appear to be affected by emigration and because the age-1+ index has more values than other postjuvenile indices[20].

### The Model

We projected the number of age-1+ Hudson River striped bass using an age-structured Leslie matrix model with random temporal variation in survival and fecundity. Age specific rates of natural mortality ($M_x$) were 1.12 for fish of ages 1+ and 2+[21]. For ages 3+ and older, natural mortality was assumed constant at an average value of $M_x$ = 0.15. Age-specific values of fecundity are shown in Table 1[21]. Recruitment (R) to age-1+ was assumed to follow Beverton-Holt type density dependence

$$R = \frac{1}{r + \frac{k}{\sum_a f_a N_a}}$$

where $f_a$ is the average fecundity of fish at age $a$ and $N_a$ is the number of fish at age $a$. The values for parameters $r$ (6.94E-04) and $k$ (4.82E+04) were derived from the relationship between the abundance of age-1+ fish and post yolk-sac larvae (Fig. 1)[17].

In our simulations, annual values of survival and fecundity were independent log-normal random variables. Values for each were obtained by multiplying the mean by

$$e^{v \ln\left(CV^2+1\right)^{1/2} - \frac{1}{2}\ln\left(CV^2+1\right)}$$

where $v$ is a standard normal random variate and $CV$ is the coefficient of variation[21]. The $CV$s for fecundity and survival for all age classes were 0.34 and 0.321, respectively[17]. The estimation of variance in survival to age 1 is described in the section on measurement error.

**TABLE 1**
**Estimates of Gear Selectivity, Fraction of Fish of Legal Size, and Fecundity (Number of Eggs per Mature Female) by Age***

| Age Class | Gear Selectivity | Fraction Legal | Mean Fecundity at Age | Initial Age Distribution |
|---|---|---|---|---|
| 1 | 0.00 | 0.08 | 0 | 1,207,500 |
| 2 | 0.02 | 0.08 | 0 | 393,800 |
| 3 | 0.14 | 0.08 | 0 | 128,100 |
| 4 | 0.43 | 0.08 | 11,190 | 109,300 |
| 5 | 0.65 | 0.08 | 111,150 | 92,900 |
| 6 | 0.84 | 0.14 | 411,680 | 78,600 |
| 7 | 0.93 | 0.46 | 747,000 | 65,400 |
| 8 | 0.97 | 0.69 | 1,083,950 | 50,400 |
| 9 | 0.98 | 0.93 | 1,451,120 | 36,600 |
| 10 | 0.99 | 0.97 | 1,676,160 | 25,100 |
| 11 | 1.00 | 0.99 | 2,022,000 | 16,900 |
| 12 | 1.00 | 1.00 | 2,301,000 | 11,400 |
| 13 | 1.00 | 1.00 | 2,285,000 | 7,600 |
| 14 | 1.00 | 1.00 | 2,342,000 | 5,100 |
| 15+ | 1.00 | 1.00 | 2,591,000 | 10,400 |

* Also shown is the initial age distribution used in model simulations.

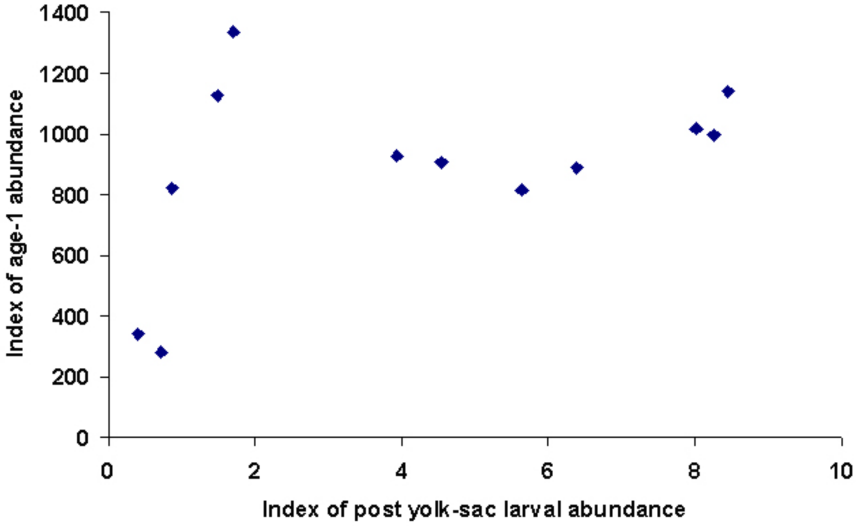

**FIGURE 1.** Recruitment, as measured by an index of age-1+ abundance, vs. stock size, as measured by an index of post-yolk-sac larval abundance, for the Hudson River stock of striped bass from 1984 through 1995, i.e., the years when values are available for both indices.

### Entrainment Mortality

Annual rates of entrainment and impingement were included in survival to age 1+. The average survival of entrainment was calculated as

$$S = \sum_{i}^{n} (1\text{-}M_i) / n$$

where $M_i$ is the mortality rate in year $i$ due to entrainment, and n is the the number of years for which data were available. The expected number of recruits to age 1+ when the effects of entrainment mortality were included was given by $R*S_E$. This method for calculating S will produce estimates that are biased low if the mortality between post-yolk-sac larvae and age-1+ fish is nonlinear due to density dependence.

The annual conditional entrainment mortality rate (CEMR) due to the operation of the Bowline Point, Indian Point, and Roseton power plants was about 11% on average from 1974 through 1997[17]. To assess the relative effect of a CEMR of 11% on recruitment, we also ran the model using a CEMR of 0%.

### Fishing Mortality

To compare the effects of fishing and entrainment, simulations were also conducted, which included age-specific estimates of fishing mortality. These were obtained by multiplying the assumed population rate of fishing mortality by an estimate of gear selectivity for each age and the fraction of fish of legal size in each age class. Specifically, the survival of each age class ($S_a$) was calculated as

$$S_a = e^{-\left[M_a + Fg(a)l(a)\right]}$$

where $M$ and $F$ are the natural and fishing mortality rates, $g(a)$ is the age specific gear selectivity, and l(a) is the fraction of fish of legal size in each age class (Table 1).

Under Amendment #5 to the Interstate Fishery Management Plan for Atlantic Striped Bass, the interim target for fishing mortality is an $F$ of 0.33 for the recovering stock and an F of 0.4 for the recovered stock. We used an $F$ of 0.33 in combination with a CEMR of 11% to represent current conditions. For comparison with a management action recommended by Amendment #5, we used an $F$ of 0.4 and a CEMR of 11%.

### Measurement Error

Measurement error represents the uncertainty in the estimates of abundance for age-1+ striped bass. Natural variability, also referred to as process error[22], reflects year-to-year changes in the conditions for survival of striped bass from juvenile (i.e., age 0) to age 1+. Partitioning natural variability from measurement error requires at least two independent, empirical estimates of abundance for age-1+ striped bass generated during the same time period; something unavailable for age-1+ striped bass. As a surrogate, we used an estimate of measurement error calculated from estimates of abundance for juvenile striped bass in the Hudson River. The location, time, and gear used to generate the abundance estimates for juvenile striped bass are different from those used to generate the estimates for age-1+ striped bass. Although the estimate of measurement error for juvenile abundance was not directly comparable, we used it to select an initial upper limit for analysis of recruitment to age 1+.

Estimates of abundance for juvenile striped bass have been calculated from two beach seine survey programs conducted on the Hudson River: the Juvenile Striped Bass Survey (JSBS) conducted by the New York State Department of Environmental Conservation and the Beach Seine Survey (BSS) conducted on behalf of four electric utilities. The JSBS sampled on alternate weeks from August through November between river-miles 25 and 40 using a 200-ft beach seine. The BSS sampled on alternate weeks from June through October along the entire length of the Hudson River using a 100-ft beach seine[17].

We represented the fraction of the population sampled by each beach seine survey as

$$\mathrm{BSS_y} = q_y R_y$$
and
$$\mathrm{JSBS_y} = p_y R_\mathrm{y}$$

where $R_y$ is the real abundance of age-0 striped bass in year $y$, while $q$ and $p$ are the proportions of the population caught by the BSS and JSBS, respectively. While the surveys are expected to sample a constant fraction of the population on average, the proportions vary each year due to measurement error. We used a log transform to obtain additive errors:

$$\ln(\mathrm{BSS_y}) = \ln(q_y) + \ln(R_y)$$
and
$$\ln(\mathrm{JSBS_y}) = \ln(p_y) + \ln(R_y)$$

If there is no covariance between annual fluctuations in $\ln(R)$ and measurement errors in $p$ and $q$, the variance in each index is

$$\mathrm{Var}(\ln(\mathrm{BSS})) = \mathrm{Var}(\ln(q)) + \mathrm{Var}(\ln(R))$$
and
$$\mathrm{Var}(\ln(\mathrm{JSBS})) = \mathrm{Var}(\ln(p)) + \mathrm{Var}(\ln(R))$$

If there is no covariance between the measurement errors across indices, the covariance in the log transformed indices may be used as an estimate of the interannual variability in ln($R$), i.e.,

$$\mathrm{Cov}(\ln(\mathrm{BSS}), \ln(\mathrm{JSBS})) = \mathrm{Var}(\ln(R))$$

The variance due to measurement error may then be estimated by subtracting this covariance from the variance in each index. This general approach has been used for abundance estimates[23,24] and estimates of survival[25].

A portion of the total variance in juvenile abundance is due to changes in reproductive effort among years. Reproductive effort for the Hudson River stock of striped bass, as measured by an index of post-yolk-sac larval abundance[20], was considerably different during the years from 1989 through 1997, compared with the years from 1976 through 1988. To avoid the potential confounding effect of changing reproductive effort, we only considered the years from 1989 through 1997 in our analysis (Fig. 2).

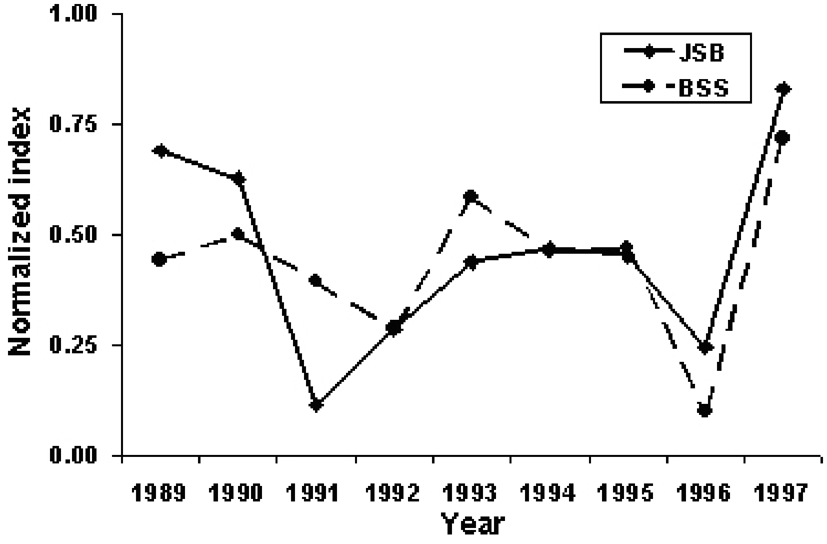

**FIGURE 2.** Indices of abundance for juvenile striped bass in the Hudson River, normalized to the highest value, caught in the Beach Seine Survey (BSS) and the Juvenile Striped Bass Survey (JSBS) from 1989 through 1997.

We used the covariance in log-transformed indices as the estimate of annual variability in abundance of age-1+ fish. Estimates of measurement error for each each index were then calculated by subtracting this estimate from the total observed variance. This method of partitioning measurement error and natural variability assumes that measurement errors are neither correlated with the actual abundance nor across indices. Measurement error could be correlated with abundance if the proportion of the population sampled varies with population size. For example, an aggregation of fish in a particular location tends to cause the proportion of fish sampled to increase as the population decreases. This is not likely the case for the indices we used because sampling is done at multiple locations during a time of the year when juvenile striped bass are migrating downriver. Furthermore, the index values we used came from years when the population was increasing in size.

Measurement error could be correlated across indices if the capture rate of juveniles by the BSS and the JSBS positively covaried because of common environmental conditions or the fraction of juveniles sampled by each index was influenced by migration among the sampling sites. This is not likely because the sampling dates and locations for the BSS and JSBS are different. The BSS covers the entire Hudson River, while the JSBS covers the lower part of the river.

Measurement error accounted for 50% of the log transformed variance in the BSS index and 56% of the log transformed variance in the JSBS index, for an average of 53%(Table 2). These estimates are comparable to analogous estimates for other species[25]. Therefore, we used 50% of the observed variability as the upper bound for measurement error. We used 0% as the lower bound and considered values of 10, 20, 30, and 40% to assess the sensitivity of risk to measurement error.

The assumed level of measurement error was subtracted from the total variance in age-1+ survival to arrive at an estimate of interannual, natural variability. To reflect the uncertainty associated with the estimate of the mean

**TABLE 2**
**Total Variance, Covariance, Variance Due to Measurement Error, and Standard Error for the Log-Transformed Beach Seine Survey (BSS) and Juvenile Striped Bass (JSBS) Indices of Striped Bass Abundance in the Hudson River during the Period 1989 through 1997**

| | Value | |
|---|---|---|
| **Statistic** | **ln(BSS)** | **ln(JSB)** |
| Total variance | 0.3360 | 0.3783 |
| Covariance | 0.1664 | 0.1664 |
| Variance due to measurement error | 0.1696 | 0.2119 |
| Standard error | 0.1373 | 0.1534 |

survival rate, upper and lower bounds were generated for each level of measurement error by performing two additional simulations. The upper bound was based on the the estimate of natural variability plus one standard error. The lower bound was based on the natural variability minus one standard error. The standard error for mean survival (Table 2) was calculated as

$$S.E. = \sqrt{\frac{Total\ Variance * \%Measurement\ Error}{n}}$$

where $n$ is the number of years of data used to estimate the mean survival rate ($n = 9$).

### Risk of a Decline in Recruitment

For each combination of measurement error, CEMR, and F, we ran the model 1,000 times for a period of 1, 5, 10, and 15 years starting from the equilibrium age distribution. The 1-year period was the smallest whole-year increment we could examine. The 5-year period corresponded to the length of a SPDES permit. The 10-year period represented issuance of two consecutive SPDES permits, a condition of the Hudson River Cooling Tower Settlement Agreement[26]. The 15-year period corresponded to issuance of three consecutive SPDES permits and the number of years remaining before the license issued by the Nuclear Regulatory Commission for the Indian Point 3 Nuclear Power Plant expired.

Ecological risk assessments are conducted to evaluate the likelihood of adverse ecological effects and not simply to calculate the probability of a common ecological occurrence. Therefore, we wanted to use a rare event as our criterion for calculating risk. The criterion we selected was unusually low recruitment expressed as a probability equal to the proportion of 1,000 model runs in which age-1+ abundance falls below 20% of the initial value at least once during the time period selected. The 20% threshold corresponds to the approximate difference between the lowest and highest estimates in the index of age-1+ abundance for Hudson River striped bass during the period from 1984 through 1997. Although other threshold values could have been selected, there are no established criteria. So rather than selecting a threshold arbitrarily, we used empirical data. Regardless of the value selected, the relative effect of measurement error would be the same.

### Analysis

We tested the effect of measurement error and time on recruitment using a nonparametric Friedman rank sums test[27].

## RESULTS

### Effect of Time Horizon

Time horizon had a significant effect on the probability that recruitment of age-1+ striped bass in the Hudson River stock would fall below 20% of the initial value ($p = 0.0007$ with a CEMR of 0% and $p = 0.0004$ with a CEMR of 11%). This effect was most pronounced when all of the variation was assumed to be natural (Figs. 3 and 4). Under this condition, the probability that recruitment

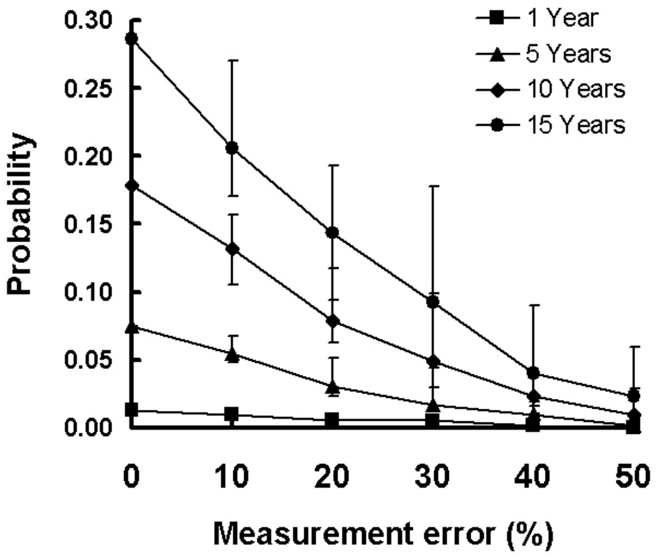

**FIGURE 3.** Probability that recruitment of age-2 striped bass in the Hudson River stock would fall below 20% of the initial value at least once for levels of measurement error ranging from 0 to 50% of the observed variability after 1, 5, 10, and 15 years with a conditional entrainment mortality rate of 0% and an F of 0.33. The bounds for each estimate are based upon simulations involving the specified level of measurement error plus and minus one standard error for mean survival. When measurement error was 0%, the bounds were so small that they were not displayed.

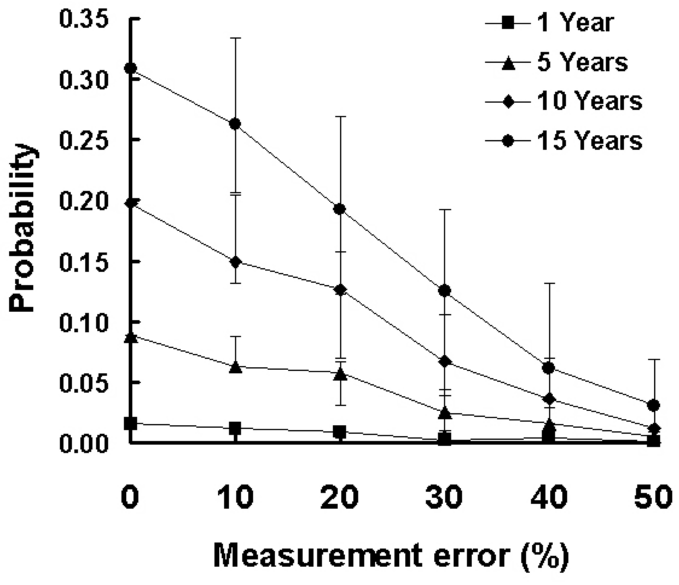

**FIGURE 4.** Probability that recruitment of age-2 striped bass in the Hudson River stock would fall below 20% of the initial value at least once for levels of measurement error ranging from 0 to 50% of the observed variability after 1, 5, 10, and 15 years with a conditional entrainment mortality rate of 11% and an F of 0.33. The bounds for each estimate are based upon simulations involving the specified level of measurement error plus and minus one standard error for mean survival. When measurement error was 0%, the bounds were so small that they were not displayed.

would fall below the 20% threshold was less than 0.016 after 1 year. After 5 years it increased to 0.075 with a CEMR of 0% and to 0.090 with a CEMR of 11%. After 10 years, it more than doubled. Over a 15-year period, the probability that recruitment would fall below the 20% threshold was more than three times higher than for a 5-year period with a CEMR of 11% (0.308) and about 4 times higher than for a 5-year period with a CEMR of 0% (0.287).

### Effect of Measurement Error

The range of measurement errors evaluated in this study had a significant effect on the probability that recruitment would fall below the 20% threshold ($p$ = 0.0015 with a CEMR of 0% and a CEMR of 11%). A measurement error of 50%, the empirically derived estimate from the abundance measures for juvenile striped bass, caused a large reduction in the probability that recruitment would fall below the 20% threshold. Over a 15-year period, the probability that

**TABLE 3**
**The Probability that Recruitment of Age-1+ Striped Bass in the Hudson River Stock would Fall below 20% of the Initial Value at Least Once after 1, 5, 10, and 15 Years by Level of Measurement Error, Fishing, and Conditional Entrainment Mortality Rate (CEMR)**

| | | Probability | | |
|---|---|---|---|---|
| | Measurement | F = 0.33 | | F = 0.4 |
| Years | Error | CEMR = 0% | CEMR = 11% | CEMR = 11% |
| 1 | 0 | 0.013 | 0.016 | 0.016 |
| 1 | 10 | 0.010 | 0.012 | 0.015 |
| 1 | 20 | 0.006 | 0.010 | 0.014 |
| 1 | 30 | 0.001 | 0.003 | 0.005 |
| 1 | 40 | 0.002 | 0.005 | 0.001 |
| 1 | 50 | 0.000 | 0.002 | 0.001 |
| 5 | 0 | 0.075 | 0.090 | 0.110 |
| 5 | 10 | 0.055 | 0.064 | 0.076 |
| 5 | 20 | 0.031 | 0.059 | 0.055 |
| 5 | 30 | 0.017 | 0.026 | 0.027 |
| 5 | 40 | 0.010 | 0.016 | 0.008 |
| 5 | 50 | 0.002 | 0.005 | 0.008 |
| 10 | 0 | 0.179 | 0.198 | 0.231 |
| 10 | 10 | 0.132 | 0.150 | 0.176 |
| 10 | 20 | 0.079 | 0.127 | 0.122 |
| 10 | 30 | 0.049 | 0.068 | 0.073 |
| 10 | 40 | 0.023 | 0.036 | 0.033 |
| 10 | 50 | 0.010 | 0.013 | 0.018 |
| 15 | 0 | 0.287 | 0.308 | 0.384 |
| 15 | 10 | 0.206 | 0.263 | 0.280 |
| 15 | 20 | 0.144 | 0.192 | 0.210 |
| 15 | 30 | 0.093 | 0.125 | 0.133 |
| 15 | 40 | 0.040 | 0.062 | 0.072 |
| 15 | 50 | 0.023 | 0.032 | 0.038 |

recruitment would fall below the 20% threshold was 0.023 with a measurement error of 50% and CEMR of 0% and was 0.032 with a measurement error of 50% and a CEMR of 11%. These are one-tenth the values with a measurement error of 0% (Table 3). For periods less than 15 years, the probability that recruitment would fall below the 20% threshold with a measurement error of 50% was substantially less than that observed with a measurement error of 0%.

The error bounds on the risk of an 80% decline in recruitment overlapped, after 10 and 15 years, with a measurement error of 20% or higher. The error bounds for all time periods overlapped with a measurement error of 40% or higher.

### Comparison of CEMR and Fishing

With an F of 0.33, CEMR had a small effect on the probability that recruitment would fall below the 20% threshold. Over a 15-year period, an increase in the CEMR from 0 to 11% increased the probability that recruitment would fall below the 20% threshold from 0.287 to 0.308 with a measurement error of 0% and from 0.023 to 0.032 with a measurement error of 50% (Table 3). With a CEMR of 11%, an increase in F from 0.33 to 0.40 had a small effect on the probability that recruitment would fall below the 20% threshold. The increase in the probability that recruitment would fall below the 20% threshold, averaged across all levels of measurement error, was only 0.023 over a 15-year period (Table 3).

## DISCUSSION

The results from this study are consistent with the conclusions of Ginzburg and Ferson[5]. Measurement error at the level generated from indices of abundance for juvenile striped bass in the Hudson River (50%) had a significant effect on risk. When all of the variation was assumed to be natural and, thus, there was no measurement error, the probability that recruitment would fall below the 20% threshold overestimated risk about tenfold after 15 years. Overestimates of this magnitude could produce conservative impact assessments and require costly efforts to reduce entrainment mortality that may not measurably reduce risk.

Accurate estimates of risk are necessary but not sufficient for defining AEI. A change in risk should be related to a previously established benchmark, such as the one provided by Amendment #5 to the Interstate Fishery Management Plan for Atlantic striped bass. Amendment #5 recommended an increase in F from 0.33 to 0.40, which had about the same effect on the risk of a decline in recruitment to the Hudson River stock of striped bass as an increase in CEMR from 0 to 11%. Thus, if sustainability of the Hudson River stock of striped bass is not reduced by the change in risk associated with the increased fishing mortality, it is not reduced by the change in risk associated with entrainment.

It is important to know if sustainability of the Hudson River stock of striped bass would be reduced if consecutive SPDES permits were issued to the Bowline, Indian Point, and Roseton power plants for a period of up to 15 years.

Although risk increases with time, the differences in risk among time horizons of 5, 10, or 15 years were smaller than the uncertainty associated with the estimates of risk when measurement error was equal to 50%. If the estimate of measurement error based on juvenile striped bass in the Hudson River (50%) corresponds to the level of measurement error for age-1+ fish, consecutive discharge permits should not reduce sustainability of the Hudson River stock.

### ACKNOWLEDGMENTS

The New York Power Authority and EPRI (Electric Power Research Institute) of Palo Alto, CA, supported this research under contract CF6871-001-12191.

## References

1. U.S. Environmental Protection Agency (1977) Interagency 316(a) Technical Guidance Manual and Guide for Thermal Effects Sections of Nuclear Facilities Environmental Impact Statements. Office of Water Enforcement, Permits Division, Industrial Permits Branch, Washington, D.C.
2. Foster, K.R., Vecchia, P., and Repacholi, M.H. (2000) Science and the precautionary principle. *Science* **288,** 279–371.
3. Nagle, D.G. and Morgan, J.T., Jr. (2000) A draft regulatory framework for analyzing potential adverse environmental impact from cooling water intake structures. *Environ. Sci. Policy* **4**, 1–8, ix–xiv.
4. U.S. Environmental Protection Agency (1998) Guidelines for ecological risk assessment. *Federal Register* **63**, 26846–26923.
5. Ferson, S. and Ginzburg, L.R. (1996) Different methods are needed to propagate ignorance and variability. *Reliab. Eng. Sys. Safe.* **54**, 133–144.
6. Stephan, T. and Wissel, C. (1999) The extinction risk of a population exploiting a resource. *Ecol. Model.* **115**, 217–225.
7. Foley, P. (1994) Predicting extinction times from environmental stochasticity and carrying capacity. *Conserv. Biol.* **8**, 124–137.
8. Lande, R. (1993) Risks of population extinction from demographic and environmental stochasticity and random catastrophes. *Am. Nat.* **142**, 911–927.
9. Pimm, S.L., Jones, H.L., and Diamond, J. (1988) On the risk of extinction. *Am. Nat.* **132**, 757–785.
10. Ludwig, D. and Walters, C.J. (1981) Measurement errors and uncertainty in parameter estimates for stock and recruitment. *Can. J. Fish. Aquat Sci.* **38**, 711–720.
11. Walters, C.J. and Ludwig, D. (1981) Effects of measurement errors on the assessment of stock-recruitment relationships. *Can. J. Fish. Aquat. Sci.* **38**, 704–710.
12. Rosenberg, A.A. and Restrepo, V.R. (1994) Uncertainty and risk in stock assessment advice for U.S. Marine Fisheries. *Can. J. Fish. Aquat. Sci.* **51**, 2715–2720.
13. Barnthouse, L.W., Boreman, J., Christensen, S.W., Goodyear, C.P., Van Winkle, W., and Vaughn, D.S. (1984) Population biology in the courtroom: the Hudson River controversy. *BioScience* **34**, 14–19.
14. Barnthouse, L.W., Klauda, R.J., and Vaughn, D.S. (1988) Introduction to the monograph. *Am. Fish. Soc. Monogr.* **4**, 1–8.
15. Dunning, D.J., Ross, Q.E., and Merkhofer, M.W. (2000) Multiattribute utility analysis for addressing section 316(b) of the Clean Water Act. *Environ. Sci. Policy* **4**, S7–S14.

16. Colquhoun, J. (2000) Hudson River Estuary Management Advisory Committee 31 January 2000 briefing by NYSDEC on: Status of the Hudson River Settlement Agreement Power Plant regulation (SPDES and EIS). New York State Department of Environmental Conservation, Albany.
17. Central Hudson Gas & Electric Corp., Consolidated Edison Company of New York, Inc., New York Power Authority, and Southern Energy New York (1999) Draft Environmental Impact Statement for State Pollutant Discharge Elimination System Permits for Bowline Point 1 & 2, Indian Point 2 & 3, and Roseton 1& 2 Steam Electric Generating Stations. New York State Department of Environmental Conservation, Albany.
18. Goldwasser, L., Ferson, S., and Ginzburg, L. (2000) Variability and measurement error in extinction risk analysis: the northern spotted owl on the Olympic Peninsula. In *Quantitative Methods for Conservation Biology*. Ferson, S. and Burgman, M., Eds. Springer-Verlag, New York. pp. 169–187.
19. Atlantic States Marine Fisheries Commission (1975) Amendment #5 to the Interstate Fishery Management Plan for Atlantic Striped Bass. Atlantic States Marine Fisheries Commission, Washington, D.C.
20. Ross, Q.E., Dunning, D.J., Young, J., and Heimbuch, D.G. Impact of entrainment on recruitment of Hudson River striped bass: an empirical approach, in preparation.
21. Saila, S., Martin, B., Ferson, S., Ginzburg, L., and Millstein, J. (1991) Demographic modeling of selected fish species with RAMAS. EPRI (Electric Power Research Institute) Palo Alto, CA, EPRI Research Project 2553, EN-7178.
22. Hilborn, R. and Mangel, M. (1997) *The Ecological Detective: Confronting Models with Data*. Princeton University Press, Princeton.
23. Skalski, J.R. and Robson, D.S. (1992) *Techniques for Wildlife Investigations: Design and Analysis of Capture Data*. Academic Press, San Diego.
24. Link, W.A. and Nichols, J.D. (1994) On the importance of sampling variance to investigations of temporal variation in animal population size. *Oikos* **69**, 539–544.
25. Gould, W.R. and Nichols, J.D. (1998). Estimation of temporal variability of survival in animal populations. *Ecology* 7**9**, 2531–2538.
26. Barnthouse, L., Boreman, J., Englert, T.L., Kirk, W.L., and Horn, E.G. (1988) *Am. Fish. Soc. Monogr.* **4**, 267–273.
27. Sokal, R.R. and Rohlf, F.K. (1969) *Biometry*. W.H. Freeman, San Francisco.
